# Metabolic Syndrome and Salt-Sensitive Hypertension in Polygenic Obese TALLYHO/JngJ Mice: Role of Na/K-ATPase Signaling

**DOI:** 10.3390/ijms20143495

**Published:** 2019-07-16

**Authors:** Yanling Yan, Jiayan Wang, Muhammad A. Chaudhry, Ying Nie, Shuyan Sun, Jazmin Carmon, Preeya T. Shah, Fang Bai, Rebecca Pratt, Cameron Brickman, Komal Sodhi, Jung Han Kim, Sandrine Pierre, Deepak Malhotra, Gary O. Rankin, Zi-jian Xie, Joseph I. Shapiro, Jiang Liu

**Affiliations:** 1 Departments of Clinical & Translational Sciences, Biomedical Sciences, and Medicine, Joan C. Edwards School of Medicine, Marshall University, Huntington, WV 25755, USA; 2 Hebei Medical University, Shijiazhuang 50017, China; 3 Department of Medicine, The University of Toledo College of Medicine and Life Sciences, Toledo, OH 43614, USA

**Keywords:** Na/K-ATPase, salt-sensitive hypertension, obesity, metabolic syndrome, pressure-natriuresis curve, reactive oxygen species

## Abstract

We have demonstrated that Na/K-ATPase acts as a receptor for reactive oxygen species (ROS), regulating renal Na^+^ handling and blood pressure. TALLYHO/JngJ (TH) mice are believed to mimic the state of obesity in humans with a polygenic background of type 2 diabetes. This present work is to investigate the role of Na/K-ATPase signaling in TH mice, focusing on susceptibility to hypertension due to chronic excess salt ingestion. Age-matched male TH and the control C57BL/6J (B6) mice were fed either normal diet or high salt diet (HS: 2, 4, and 8% NaCl) to construct the renal function curve. Na/K-ATPase signaling including c-Src and ERK1/2 phosphorylation, as well as protein carbonylation (a commonly used marker for enhanced ROS production), were assessed in the kidney cortex tissues by Western blot. Urinary and plasma Na^+^ levels were measured by flame photometry. When compared to B6 mice, TH mice developed salt-sensitive hypertension and responded to a high salt diet with a significant rise in systolic blood pressure indicative of a blunted pressure-natriuresis relationship. These findings were evidenced by a decrease in total and fractional Na^+^ excretion and a right-shifted renal function curve with a reduced slope. This salt-sensitive hypertension correlated with changes in the Na/K-ATPase signaling. Specifically, Na/K-ATPase signaling was not able to be stimulated by HS due to the activated baseline protein carbonylation, phosphorylation of c-Src and ERK1/2. These findings support the emerging view that Na/K-ATPase signaling contributes to metabolic disease and suggest that malfunction of the Na/K-ATPase signaling may promote the development of salt-sensitive hypertension in obesity. The increased basal level of renal Na/K-ATPase-dependent redox signaling may be responsible for the development of salt-sensitive hypertension in polygenic obese TH mice.

## 1. Introduction

Obesity, hypertension and type 2 diabetes, as a group of risk factors, are significant causes of morbidity and mortality throughout the world. Although these disorders often cluster in individuals, the basis for this aggregation is not well understood [1]. Hypertension is a key modifiable risk factor. Moreover, obesity and metabolic syndrome are phenotypes that are strongly associated with salt-sensitive hypertension in preclinical obesity and metabolic syndrome animal models, and very likely in humans as well [2]. When considering the growing worldwide epidemic of obesity and metabolic syndrome and the constellation of comorbidities that complicate their management, the burden on the healthcare system is tremendous. Therefore, there is an urgent need to investigate the mechanisms linking obesity and hypertension and to develop appropriate and effective therapeutic regimens, preventing and limiting obesity-related metabolic disorders and hypertension [3]. 

The best known mechanisms of salt-sensitive hypertension in obesity and metabolic syndrome are altered hemodynamics, impaired Na^+^ homeostasis, renal dysfunction, autonomic nervous system imbalance, endocrine alterations, oxidative stress and inflammation, and vascular injury [4]. These factors integrate interdependently to regulate salt transport in the kidney, leading to hypertension and enhanced salt sensitivity. Based on Dr. Arthur Guyton’s quantitative mathematical model, the final common pathway for engendering hypertension is decreased renal salt excretion, which expands the blood volume and increases systemic blood pressure (BP) to excrete excess salt to achieve steady-state [4,5,6,7,8]. The mechanisms of salt-sensitive hypertension continue to be contentious and the subject of considerable interest, especially in obesity and metabolic syndrome due to the risk factor clustering [8,9]. Therefore, improving our understanding of the primary etiologies that lead to salt-sensitive hypertension in obesity and metabolic syndrome will be crucial for preventing and treating these conditions. Obesity and metabolic syndrome-associated hypertension should be recognized as a distinctive form of hypertension, and specific considerations should apply in planning therapeutic approaches to treat obese individuals with high blood pressure. Only then can we explore precise target localization to render the required pharmacologic therapy more effective, minimizing the need for medications.

The sodium-potassium adenosine triphosphatase (Na/K-ATPase) enzyme (EC3.6.3.9.), or “sodium pump” was first identified by Dr. Skou on the crab nerve in 1957 [10]. Besides its transportation of ions, work from our laboratory provides evidence to identify its signaling function in renal proximal tubules. Specifically, reactive oxygen species can signal through the Na/K-ATPase signaling cascade and regulate renal Na^+^ handling and blood pressure [11,12,13]. We have documented this mechanism in the Sprague Dawley (SD) rat and Dahl salt resistant (DR) rat fed a high salt diet. However, this process is markedly impaired in the Dahl salt-sensitive (DS) rat [12]. The specific roles of Na/K-ATPase-signaling-mediated renal Na^+^ excretion and arterial BP modulation have not been investigated in obesity and metabolic syndrome-associated salt-sensitive hypertension. The purpose of the present investigation was to evaluate the hypothesis that a similar blunting of the pressure diuresis-natriuresis relationship is implicated in the development of hypertension in obesity and metabolic syndrome-associated salt-sensitive hypertension. 

The TALLYHO/JngJ (TH) mouse is an excellent model to mimic the state of obesity in humans with a polygenic background of type 2 diabetes [14], representing a mouse model of obesity and metabolic syndrome. Nothing is known regarding the mechanisms that mediate the potent effects of Na/K-ATPase signaling on pressure natriuresis and salt-sensitivity of blood pressure in TH mice. The first goal of the present study was to characterize high salt responses in TH mice. As a part of these studies, we examined whether TH mice developed salt-sensitive hypertension and whether impaired renal Na^+^ excretion plays a role in this model. The second goal was to examine the Na/K-ATPase signaling responses to a high salt diet in the kidneys in both B6 and TH mice and discuss the potential reasons. The results indicate that the diuretic and natriuretic responses of TH mice to elevations in arterial pressure were 32.5% and 37.9%, respectively, less than those seen in the control B6 mice when challenged with a high salt diet. Salt-sensitive hypertension correlated with changes in the Na/K-ATPase signaling.

## 2. Results

### 2.1. TH Mouse Characteristics

**Figure 1:**
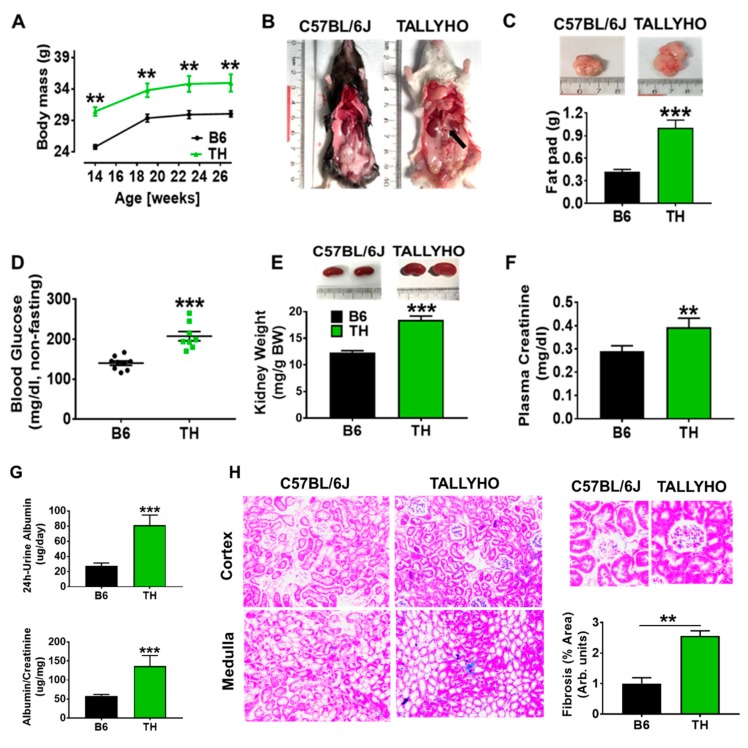
TALLYHO/JngJ (TH) mice on normal chow exhibit the risk-factor clustering and kidney dysfunction associated with obesity and metabolic disorders. TH and C57BL/6J (B6) mice were monitored for a time period of 13 weeks, starting at the age of 14 weeks. (**A**) Body mass (*n* = 8–9). (**B**) Abdominal photograph of representative 23–27 week old male mice (arrow showed the renal sinus fat). (**C**) Fat-pad mass (*n* = 7–8). (**D**) Non-fasting blood glucose levels (*n* = 8–9). (**E**) Kidney mass (*n* = 9–10). (**F**) Plasma creatinine (*n* = 7–10). (**G**) 24 h urinary albumin excretion rate and urinary albumin to creatinine ratio (*n* = 7–9). (**H**) Masson’s trichrome staining of kidneys. Representative and quantitative assessment of the areas of extracellular matrix in the glomerulus and tubulointerstitial fibrosis (original magnification, ×20) mean ± SEM of trichrome-stained photomicrographs obtained from right kidney tissues from B6 and TH mice. ** *p* < 0.01, *** *p* < 0.001 versus B6 normal chow.

Analysis of these TALLYHO/JngJ and C57BL/6J (B6) mice showed that, at 14–27 weeks of age, TH mice had body masses (body weight, BWs) that were on average 15% greater than those of wild-type (WT) mice (Figure 1A). The increased BWs in the TH groups compared with B6 groups were also mirrored by the elevated adiposity. Figure 1B,C revealed increased visceral fat mass including retroperitoneal, perirenal and epididymal fat pads that were about twice as large as those in WT B6 mice at 23–27 weeks of age. Particularly, excess fat accumulation around the kidneys, known as fatty kidneys, is shown in Figure 1B. However, no significant differences were recorded between the high salt and normal chow groups. In contrast to WT B6 mice, TH mice exhibited mild but statistically significant elevations in non-fasting blood glucose (Figure 1D). The 49.4% increase in kidney size (kidney weight/body weight) observed in obese TH mice differed significantly from the values observed in B6 mice (Figure 1E). Moreover, there was a substantial increase in both plasma creatinine (Figure 1F) and 24 h urinary albumin excretion (Figure 1G) in obese TH mice when compared to the B6 mice. Semi-quantitative data on renal histology also showed an increase in glomerular and tubule-interstitial fibrosis in TH mice (Figure 1H). These findings indicate that the risk-factor clustering for cardiovascular disease associated with obesity and metabolic syndrome may play a role in impaired renal function exhibited by the obese TH mice.

### 2.2. Systolic Blood Pressure, Renal Na^+^ Excretion, and Na/K-ATPase Signaling with Normal Chow in Obese TH Mice

**Figure 2:**
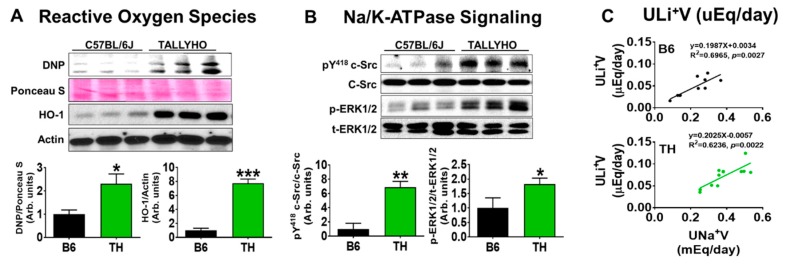
Na/K-ATPase signaling is implicated in proximal tubule Na^+^ handling and blood pressure regulation. At the age of 23-27 weeks, TH mice were compared with wild-type (WT) B6 mice. Western blots and quantification of kidney cortex tissues showed that activated baseline protein carbonylation (DNP) and heme oxygenase (HO-1) (**A**) and Na/K-ATPase signaling (c-Src and ERK1/2 phosphorylation) (**B**) were observed in obese TH mice. (**C**) Correlation of lithium excretion (ULi^+^V) and Na^+^ excretion rate (UNa^+^V) in TH and B6 mice. The correlation analysis was evaluated by Pearson correlation test. Regression line was drawn. B6: *n* = 10, *r* = 0.8346, *p* = 0.0027; TH: *n* = 12, *r* = 0.7897, *p* = 0.0022. *p* values for the regression coefficient are given. Results in (A) and (B), *n* = 4. * *p* < 0.05; ** *p* < 0.01; *** *p* < 0.001 versus B6.

The regulation of blood pressure requires the maintenance of a balance between Na^+^ and water intake and Na^+^ and water excretion. We have reported that reactive oxygen species (ROS) can signal through the Na/K-ATPase signaling cascade including Src and ERK phosphorylation [11,12,13], which is capable of regulating renal proximal tubule Na^+^ transport [11,13,15,16,17]. Impairment of Na/K-ATPase signaling contributes to salt-sensitive hypertension in the experimental rat model [12]. Table 1 and Figure 2 illustrate Na/K-ATPase signaling is involved in regular salt and water balance, leading to stable body fluid volumes and the maintenance of normal blood pressure. As shown in Table 1, there was no significant difference in systolic blood pressure or total and fractional Na^+^ excretion rate between wild-type B6 and obese TH mice. Moreover, Na/K-ATPase signaling was activated as demonstrated by increased protein carbonylation and heme oxygenase-1 (widely used markers for enhanced ROS production, Figure 2A), and phosphorylation of c-Src and ERK1/2 (Figure 2B) in the kidney cortex tissues of TH mice, in comparison to WT B6 mice. More importantly, there were significant positive correlations between Na^+^ excretion and lithium (Li^+^) excretion rate over 24 h (Figure 2C), suggesting that the proximal tubule is engaged in Na^+^ handling in both of these strains. 

### 2.3. Systolic Blood Pressure and Fluid Retention with High Salt Diet in Obese TH Mice

**Figure 3:**
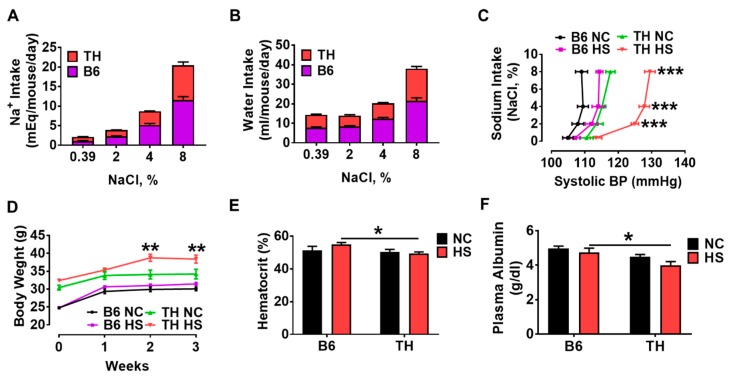
TH mice develop salt-sensitive hypertension due to blood volume expansion. Increased Na^+^ intake (**A**, *n* = 8–10) and water intake (**B**, *n* = 8–10) were observed in both B6 and TH mice. (**C**) Renal function curve was plotted with systolic blood pressure (BP) on the abscissa and the Na^+^ intake on the ordinate. Compared to the wild-type B6 mice, high salt significantly shifted the renal function curve to the right in obese TH mice (*n* = 10–12). *** *p* < 0.001 versus TH NC at different amounts of Na^+^ intake respectively. (**D**) Body weight gain (*n* = 7–8). NC, normal chow group, 0.39% NaCl; HS, high salt group, 7 days of 2% NaCl, 7 days of 4% NaCl, and then 7 days of 8% NaCl. ** *p* < 0.01 versus TH NC at different amounts of Na^+^ intake respectively. (**E**) Hematocrit (*n* = 9–13). * *p* < 0.05. (**F**) Plasma albumin (*n* = 8). * *p* < 0.05.

Although the TH mice have similar normal levels of systolic blood pressure (SBP) compared to B6 mice fed with normal chow ([14,18] and Table 1), to the best of our knowledge, we were the first to demonstrate that TH mice were hypertensive in terms of salt sensitivity based on the renal functional curve studies. Weight stable and weight gaining data (Figure 3D) showed that both B6 and TH mice had enough food intake. When quantifying the differences in food and Na^+^ consumption, at least two factors should be taken into consideration: (1) overestimation of actual food and Na^+^ consumption resulting from food spilling, since we observed that B6 mice are more active and drop more food into the bedding during feeding than TH mice; (2) how long it takes to get acclimatized to high salt diets. Therefore, to minimize the variations caused by the factors mentioned above, we compared the Na^+^ intake throughout the entire experiment using the self-comparison (the increases in 24 h Na^+^ intake in Table 2). If assuming that both B6 and TH mice dropped food into the beddings in the same rate across the experiment, the Na^+^ intake was increased by 1.173-fold at 2% NaCl (compared to normal chow), 1.311-fold at 4% NaCl (compared to 2% NaCl) and 1.316-fold at 8% NaCl (compared to 4% NaCl) in B6 mice; the TH mice had a rise in Na^+^ consumption by 0.607-fold at 2% NaCl, 1.218-fold at 4% NaCl and 1.541-fold at 8% NaCl (Table 2). This suggested that the TH mice might take a little longer time to get adapted to gradually increased high salt intake. Thus, it would be reasonable to interpret the differences in the renal handling of Na^+^ throughout the entire experiment. 

**Figure 4:**
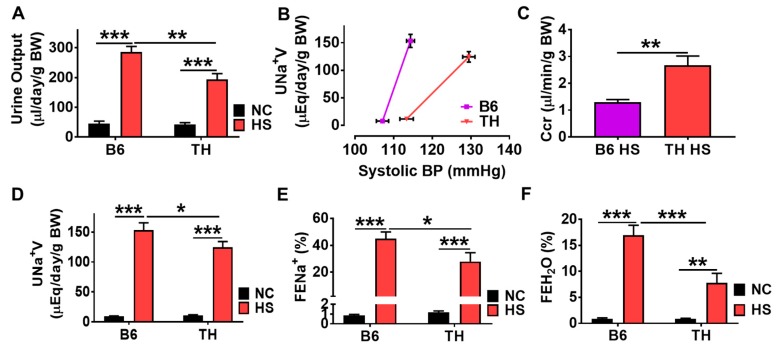
TH mice encounter blunted renal Na^+^ excretion. (**A**) Urine output (*n* = 8–10). (**B**) Pressure-natriuresis curve was plotted with systolic BP on the abscissa and the total Na^+^ excretion on the ordinate (*n* = 7–9). (**C**) Creatinine clearance (Ccr, *n* = 7–9). (**D**) Total Na^+^ excretion rate (*n* = 6–7). (**E**) Fractional Na^+^ excretion (*n* = 8–10). (**F**) Fraction water excretion (*n* = 7–9). Results in A–F, * *p* < 0.05; ** *p* < 0.01; *** *p* < 0.001. Urinary excretion data were all factored per gram body weight. HS, 7 days of 2% NaCl, 7 days of 4% NaCl, and then 7 days of 8% NaCl.

High dietary salt intake presents a major challenge to the kidneys to excrete large amounts of salt administered. On normal chow, the TH mice appeared to be borderline hypertensive. At the end of the 2% NaCl diet, the increases in Na^+^ intake in TH (0.607 ± 0.366) were less than B6 mice (1.173 ± 0.627, Table 2). However, TH mice showed a significant rise in systolic blood pressure (Figure 3C), further suggesting that TH mice were salt-sensitive hypertensive. At the end of the experiment, even if the increases in Na^+^ consumption were a little, but not significantly, more in TH mice (1.541 ± 0.759) as compared to B6 mice (1.316 ± 0.562, Table 2), the TH mice showed an impairment of renal Na^+^ handling (Figure 4A,D–F), resulting in an increase in systolic blood pressure (Figure 3C and Figure 4B) in comparison to the wild-type B6 mice. Essentially, as Na^+^ and water intake increased (Figure 3A,B, Table 2), the renal function curve of TH mice was not only shifted to the right, but its slope was also decreased (Figure 3C) as compared to the normal chow group, indicating that TH mice exhibited a more sensitive response to a high salt intake regarding systolic blood pressure.

Salt-induced hypertension is initiated by increased blood volume. Total peripheral resistance (TPR) then becomes elevated, responsible for hypertension [8,19,20,21]. The volume of plasma and blood has been derived from indirect estimates of changes in the concentration of plasma proteins and the hematocrit [19,20,21]. We next explored hemodynamic mechanisms initiating hypertension on a high salt diet in obese TH mice. After three weeks of a high salt diet, body weight in the TH groups on both high and regular salt diets was significantly higher than those in control groups (Figure 3D). High salt diets did not result in increased food consumption in TH mice on the respective diets versus their counterparts, reflecting that TH mice comsumed a similar amount of food as the B6 mice. Therefore, weight gain of about 4 g within 3 weeks of high salt diet (Figure 3D) in TH mice is not due to hyperphagia, suggesting fluid retention. At this moment, we believe that on normal chow, the TH mice are heavier than B6 mice due to the elevated adiposity (Figure 1B,C). While on a high salt diet, obese TH mice gained weight, which suggested fluid retention because there were no significant differences in fat-pad mass recorded between the high salt and normal chow groups. As compared to the WT B6 mice, the observed fall in hematocrit and plasma albumin in response to a high salt diet in TH mice (Figure 3E,F) indicated an increase in the plasma volume, in parallel with a significant increase in body weight (Figure 3D). Therefore, hypertension in obese TH mice on a high salt diet was caused by increased blood volume and water retention.

### 2.4. Impaired Renal Na^+^ Excretion and Na/K-ATPase Signaling with High Salt Diet in Obese TH Mice

Research during the past four decades has demonstrated that obesity shifts the renal function curve to higher blood pressure due to the impaired renal Na^+^ excretion [3,22,23,24,25]. The relationships between the excretion of Na^+^ and water and blood pressure in TH and B6 mice are presented in Figure 3C and Figure 4A–F. In normal chow groups, 24 h urine output and Na^+^ excretion rate were not significantly different, averaging 45.39 μL/day/g BW versus 42.67 μL/day/g BW in urine output (Figure 4A) and 9.261 μEq/day/g BW versus 10.64 μEq/day/g BW in urine Na^+^ excretion rate (Figure 4D) for B6 and TH mice respectively. Increasing SBP by 7 mmHg in the control B6 mice (from 107 to 114 mmHg, Figure 3C and Figure 4B) led to a 5.3-fold increase in urine output as well as comparable increases in total and fractional Na^+^ excretion (15.6-fold and 52.3-fold, respectively, Figure 4A,D,E). 

In the salt-sensitive hypertensive TH mice, the diuretic and natriuretic response to changes in arterial pressure were less than those seen in the control B6 mice. Increasing SBP by 17 mmHg from 113 to 130 mmHg (Figure 3C and Figure 4B) produced a 3.5-fold increase in urine output (Figure 4A) and 10.7-fold increase in total Na^+^ excretion (Figure 4D). Similarly, there was a significant 22.6-fold rise in fractional Na^+^ excretion in the TH mice (Figure 4E) as SBP was elevated over this range. In other words, TH mice fed a high salt diet showed the rightward shift and shallow slope in the renal function curve (Figure 3C and Figure 4B), and also 32.5% and 37.9% less than B6 mice in urine output (Figure 4A) and fractional Na^+^ excretion (Figure 4E). It is evident that the pressure-diuretic and -natriuretic responses to a high salt diet in obese TH mice were diminished. A significant rise in renal water reabsorption as shown by a decreased fractional water excretion (Figure 4F) also supports this finding. We validated the observations by a high salt diet with a Na^+^ bolus and volume expansion in the anesthetic mouse. SBP significantly increased in TH mice in response to saline loading compared to B6 mice. Volume expansion caused marked increases in sodium excretion in B6 mice, but these increases were significantly attenuated in polygenic obese TH mice (Figure S1). 

**Figure 5:**
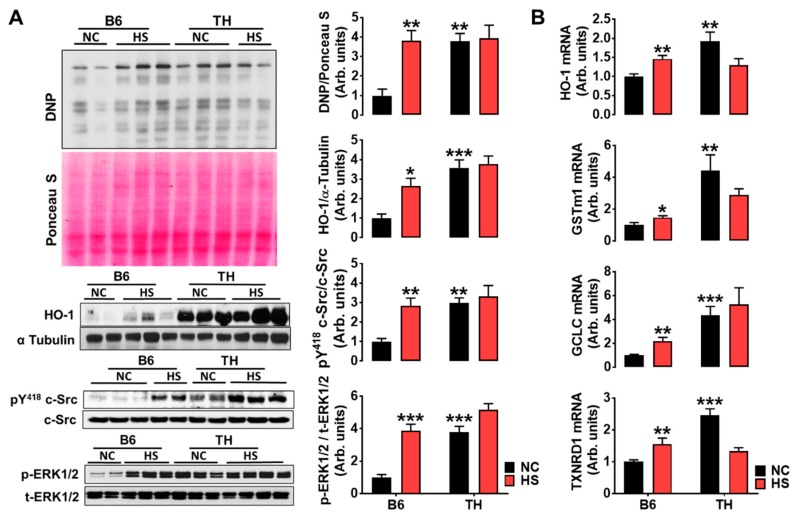
TH mice encounter dysregulation of Na/K-ATPase signaling. (**A**) In the kidney cortex tissues, reactive oxygen species (DNP: *n* = 5–10; HO-1: *n* = 9–12) and c-Src (*n* = 6–8) and ERK1/2 (*n* = 6–12) phosphorylation were activated, but not stimulated by HS diet in obese TH mice, in comparison to wild-type B6 mice. Left panel, representative Western blots. Right panel, quantitative analysis. (**B**) qRT-PCR analysis of nuclear factor erythroid 2-related factor 2 (Nrf2) target genes in the kidney cortex tissues. GSTm1, glutathione S-transferase M1. GCLC, glutamate-cysteine ligases. TXNRD1, thioredoxin reductase 1. * *p* < 0.05; ** *p* < 0.01; *** *p* < 0.001 versus B6 NC. HS, 7 days of 2% NaCl, 7 days of 4% NaCl, and then 7 days of 8% NaCl.

A significant increase in creatinine clearance (Ccr, an index of glomerular filtration rate, GFR) was observed in obese TH mice (Figure 4C), suggesting that the ability to maintain Na^+^ balance during high salt hypertension may be dependent on an elevated GFR. Regarding the molecular mechanisms for impaired pressure-natriuresis, we assessed the Na/K-ATPase signaling function in the kidney cortex tissues. Figure 5A showed that a high salt diet stimulated redox signaling and phosphorylation of c-Src and ERK1/2 in WT B6 mice. However, this phenomenon was not seen in obese TH mice. The dysfunctional Na/K-ATPase signaling seemed to be attributed to over-stimulated baseline protein carbonylation and c-Src and ERK1/2 phosphorylation when compared to their relative controls. Heme oxygenase (HO-1) expression is usually regulated by redox-sensitive transcription factor nuclear factor erythroid 2-related factor 2 (Nrf2). Consistent with the expression pattern of the HO-1 protein, the qRT-PCR analysis demonstrated that Nrf2 responsive genes were significantly upregulated by high salt in WT B6 mice but not in obese TH mice. Compared to the B6 mice with normal chow, the Nrf2 target genes appeared to be upregulated already with normal chow (Figure 5B).

## 3. Discussion

### 3.1. TH Mouse Appears to Be a Suitable Model to Research the Pathophysiology of Obesity and Metabolic Syndrome-Related Chronic Kidney Disease

In 2017, the Centers for Disease Control and Prevention (CDC) reported that more than 1 in 7 US adults were estimated to have chronic kidney disease (CKD), but 96% of them were not aware of having CKD [26]. However, CKD research is hampered by a lack of an appropriate CKD mouse model. Although many genetically modified mice have been generated on a C57BL/6J background, the biggest problem is that this model does not provide data on kidney function or proteinuria. The data in the present study indicate that the TH mouse model might be useful to investigate the pathophysiology of the human obesity and metabolic syndrome-related kidney diseases. 

According to the most widely used National Cholesterol Education Program’s Adult Treatment Panel III (NCEP: ATP III) [27] and the International Diabetes Federation (IDF) [22,28] definitions, we have shown that TH mice exhibit a phenotype reminiscent of the human metabolic syndrome. The metabolic derangements in obese TH mice include fatty kidneys (Figure 1B,C), high blood sugar (Figure 1D), and salt-sensitive blood pressure (Figure 3C and Figure 4B) that are observed in obesity in humans. The bigger kidney size (Figure 1E) as shown in humans [23,24,25] suggests kidney hypertrophy. The observed increases in 24 h proteinuria and plasma creatinine and glomerular and tubule-interstitial fibrosis (Figure 1F–H) indicate decreased kidney function. When challenged with a high salt diet, TH mice show an aberration in renal Na^+^ excretion (Figure 4A,D–F). Increased fat deposition in and around the kidneys (Figure 1B) is also related to impaired pressure natriuresis and hypertension [29]. High blood glucose and hypertension are the leading cause of CKD. Given the occurrence of single gene mutations causing obesity in humans is rare, the polygenic obese TH mice appear to be an accurate replica of either human genetics or physiology [30,31,32], representing a suitable animal model to investigate the potential diagnostic and therapeutic approach for human obesity and metabolic syndrome-related kidney diseases. 

### 3.2. Renal Na/K-ATPase Signaling Malfunction Response to Dietary Salt Contributes to Blunted Na^+^ and Water Excretion, Leading to Salt-Sensitive Hypertension in Obese TH Mice

Metabolic syndrome in human patients often exhibits increased salt sensitivity [33,34] and is thought to be salt-sensitive to blood pressure. Although one study reports that the TH mice have normal levels of systolic blood pressure compared with the wild-type B6 mice [14,18], we have first demonstrated in this report that the TH mouse has the salt sensitivity or blood pressure changes in response to a high salt diet based on the following findings. First, TH mice had a 17 mmHg rise in systolic blood pressure placed on gradually increasing salt consumption (Figure 3C and Figure 4B). Secondly, the diminished pressure diuretic–natriuretic relationship was evidenced by a rightward shift of renal function curve with a decrease in the slope (Figure 3C and Figure 4B), in contrast to the control B6 mice. Thirdly, 32.5% less in daily urine output and 37.9% less in fractional Na^+^ excretion than the wild-type B6 mice was observed in obese TH mice (Figure 4A,E). Last but not least, expanded fluid and blood volume was shown in obese TH mice by the weight gain and a reduction in both hematocrit and plasma albumin levels in response to a high salt diet (Figure 3D–F).

The mechanisms responsible for salt-sensitive hypertension in obesity and metabolic syndrome involve a multifaceted process that includes activation of the sympathetic nervous and renin-angiotensin systems [3,35,36], especially increased renal sympathetic nerve activity. Moreover, renal denervation dramatically attenuates Na^+^ retention and hypertension in experimental models of obesity [36,37,38]. However, renal denervation has not provided a clinical panacea [39,40,41]. Hence, additional molecular hypotheses need to be explored. Reactive oxygen species (ROS), a frequent accompaniment of chronic disorders, have been documented in both experimental and human salt-sensitive and/or obese hypertension [42,43]. Additionally, ROS appear to be essential initiators and maintainers of sympathetic hyperactivity [44]. We have reported that ROS can signal through the Na/K-ATPase signaling cascade [45,46,47,48,49,50], which is capable of regulating renal proximal tubule Na^+^ transport [11,13,15,16,17], to modulate blood pressure in the experimental rat model of salt-sensitive hypertension [12]. Furthermore, Na/K-ATPase acts as an amplifier for ROS [51]. pNaKtide, an antagonist specifically for Na/K-ATPase signaling, blocks the signal by which the cellular Na/K-ATPase amplifies oxidants and ameliorates obesity-related abnormalities [52,53,54]. The data in this present investigation demonstrated that high salt was unable to activate Na/K-ATPase signaling further in the obese TH mice when compared to B6 mice (Figure 5A). Due to this, TH mice could not excrete Na^+^ as much as the wild-type B6 mice did (Figure 4D,E). In other words, the absence of differences in redox signaling and phosphorylation of c-Src and ERK1/2 in TH mice between the normal chow and high salt groups in Figure 5A suggested that Na/K-ATPase signaling was dysfunctional. Thus, TH mice had less capability of excreting Na^+^, leading to salt-sensitive hypertension. Specifically, the rightward shift of the renal function curve in obese TH mice was probably due mainly to the dysfunctional Na/K-ATPase signaling-induced increase in Na^+^ reabsorption by the renal tubules, which in turn required an elevated pressure and elevated glomerular filtration rate to overcome the excess Na^+^ reabsorption and still excrete the daily intake of Na^+^. The finding (Figure 4C) that the creatinine clearance (Ccr), an index of the glomerular filtration rate, was statistically higher in the obese TH mice when challenged with a high salt diet, also supports this mechanism.

Interestingly, the observed high baseline protein carbonylation and HO-1 expression (Figure 2A) in the kidney cortex tissues of TH mice are similar to that as shown in Dahl salt-sensitive rats ([12] and unpublished data). The mass spectrometry study in our recent publication demonstrates that carbonylation modification of the Na/K-ATPase may play a critical role in renal sodium handling [16]. Therefore, we postulate that the carbonylated modification of Na/K-ATPase as seen in the kidney cortex tissues of TH mice with normal chow (Figure 2A and Figure 5A), which we would like to term the Na/K-ATPase carbonylated preconditioning, might produce resistance to Na/K-ATPase signaling, resulting in an abnormal pressure diuresis-natriuresis response as shown in Figure 4. Due to this, obese TH mice might be prone to developing hypertension when challenged with a high salt diet (Figure 3C and Figure 4B). In particular, Na/K-ATPase is highly sensitive to changes in the redox state [55,56,57]. The mechanisms of its redox sensitivity remain unclear. Most recently, Dr. Petrushanko’s group reported that inhibition of Na/K-ATPase signaling caused by ouabain in murine fibroblast cells (SC-1 cell line) under hypoxic conditions was associated with a decrease in the production of reactive oxygen species and prevention of oxidative stress [58]. S-glutathionylation of cysteine residues of the Na/K-ATPase plays an essential role in this process [55]. It would be fascinating to study further the crosstalk between the carbonylation and S-glutathionylation of Na/K-ATPase regarding Na/K-ATPase signaling-mediated renal Na^+^ handling and blood pressure regulation. 

Overall, we believe the redox signaling is required in Na/K-ATPase signaling-mediated Na^+^ handling and blood pressure regulation. Furthermore, carbonylated preconditioning of Na/K-ATPase might play a key role in impaired Na/K-ATPase signaling-mediated pressure-natriuresis.

### 3.3. Proximal Na/K-ATPase Signaling Might Be Implicated in Renal Na^+^ and Water Handling in Both B6 and TH Mice 

Numerous studies have reported the association of obesity with increased reabsorption of Na^+^ in proximal tubules [59,60,61], impaired pressure natriuresis, and then hypertension [29]. Endogenous cardiotonic steroids (CTS) in humans and rodents [57,62], can signal through Na/K-ATPase, regulating renal salt handling in the proximal tubules [11,13,16] and contributing to salt-sensitive hypertension [12,15]. Since lithium (Li) is the marker for proximal tubular delivery [63], the data in this present study that correlates urinary total Na^+^ and lithium excretion showed genuinely proportional excretion of both ions (Figure 2C), implicating identical handling of lithium and Na^+^ in the proximal tubules in these two strains. In other words, changes in urinary Na^+^ excretion are paralleled by changes in urinary Li^+^ excretion. Moreover, proximal tubular Na^+^ handling is associated with the activation of Na/K-ATPase signaling (Figure 2A,B). When challenged with a high salt diet, the obese TH mice had a reduced in vivo capacity to excrete a salt load. This natriuretic handicap is associated with an impaired Na/K-ATPase signaling activation (Figure 5A), and greater renal Na^+^ reabsorption, as determined by decreased total, fractional Na^+^ excretion, contributing to the development of salt-sensitive hypertension in this strain. Since the natriuretic handicap is demonstrable only following salt loading, it appears not to be a fixed intrinsic abnormality of TH mouse kidneys. Of note, dysfunctional Na/K-ATPase signaling could be due to the activation of baseline Na/K-ATPase signaling termed the carbonylated preconditioning. This finding is consistent with our previous report in cellular observations. The stimulated baseline c-Src phosphorylation is detected in proximal tubules isolated from DS rats [12] and in α1 Na/K-ATPase knockdown porcine proximal tubular PY-17 cells [64], as well as in Pro to Ala α1 mutant porcine proximal tubular PA cells [16]. This cellular alteration could be the cause of the higher rate of kidney Na^+^ reabsorption observed in the TH mice response to a high salt diet. We appreciate the most recent publication in *Nature Communication,* which demonstrates that Src is also regulated by redox-dependent mechanisms, further supporting our findings [65]. It would be exciting to explore the mechanisms by which obesity reduces the pool of Src-interacting Na/K-ATPase and thus increases the baseline signaling function in the future. Taken together, we postulate that salt sensitivity is the result of Na/K-ATPase signaling malfunction in the proximal tubules. Specifically, serving as the receptor for ROS, the pre-activation of the proximal tubular Na/K-ATPase signaling may, in turn, reduce the effectiveness of Na/K-ATPase signaling-mediated natriuresis, leading to kidney dysfunction, characterized by increased proximal tubular reabsorption. The insufficient Na/K-ATPase signaling-mediated natriuresis causes the shift of pressure-natriuresis to higher blood pressure, compensating the initial kidney dysfunction and returning Na^+^ excretion to match intake. The mechanisms for the desensitized Na/K-ATPase signaling-mediated renal Na^+^ handling need to be researched in the future.

#### Perspective

Abnormalities in the actions of this Na/K-ATPase signaling on Na^+^ excretion may be a significant factor responsible for the salt sensitivity of blood pressure in obese TH mice. The crosstalk between the carbonylated preconditioning and S-glutathionylation of Na/K-ATPase regarding Na/K-ATPase signaling-mediated renal Na^+^ handling and blood pressure regulation remains to be explored.

We may explore the possibility further once pharmacological manipulation of the Na/K-ATPase signaling system becomes feasible in humans.

## 4. Materials and Methods 

### 4.1. Experimental Protocol

The protocol of the study was approved by the Marshall University Institutional Animal Care and Use Committee (IACUC#: 611. Approved on 25 April 2018), and all animal care followed the National Institutes of Health (NIH) Guide for the Care and Use of Laboratory Animals. Twenty week old male TALLHO/JngJ (TH) and C57BL/6J (B6) mice were obtained from the Jackson Laboratory (Bar Harbor, Maine, USA). All animals were housed in a pathogen-free facility under a controlled environment, consisting of a regulated light cycle (on 12 h, off 12 h), temperature (21–23 °C), and humidity (40–55%). The mice were divided into two groups as follows: (1) normal chow (NC); (2) high salt diet (HS, 7 days of 2% NaCl, 7 days of 4% NaCl, and 7 days of 8% NaCl). High salt diet pellets were purchased commercially from ENVIGO, Madison, WI, USA (2% NaCl, TD. 130345; 4% NaCl, TD. 110078; 8% NaCl, TD. 07501).

**Figure 6:**
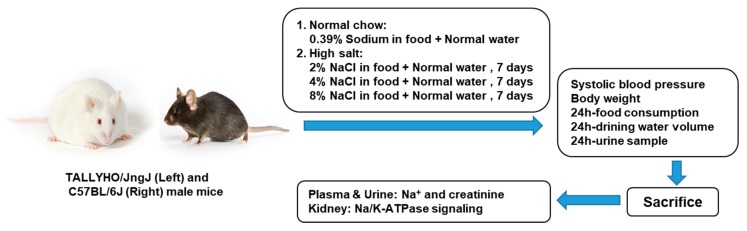
Experimental protocol. Aged-matched male obese TALLYHO/JngJ (TH) and wild-type C57BL/6J (B6) mice were divided into two groups. (1) Normal chow group: 0.39% sodium in chow; (2) High salt group: 7 days of 2% NaCl in food, 7 days of 4% NaCl in food, and then 7 days of 8% NaCl in food.

Non-fasting blood glucose levels were measured with a glucometer from submandibular bleeding. Body weight was measured every week. Blood pressure, 24 h food consumption, 24 h drinking water volume and 24 h urine sample (animal in an individual metabolic cage) were collected one day before and after the different concentrations of dietary salt. Renal function curves were used to investigate the causative mechanisms of obesity hypertension. Mice were anesthetized with the gaseous isoflurane (ISO). The induction of anesthesia was started by placement of the animal in a container with 2–3% ISO, whereas maintenance of anesthesia was provided by continuous inhalation of 1.5–2% ISO. Kidneys were obtained to test Na/K-ATPase signaling function (Figure 6). 

### 4.2. Blood Pressure Measurement

Systolic blood pressure (SBP) was monitored on conscious animals by a tail-cuff method using CODA 8-Channel High Throughout Non-Invasive Blood Pressure System (Kent Scientific, Boston, MA, USA) following at least two weeks of daily training by a single operator. To obtain a reliable SBP value from each mouse, the mean of at least ten consecutive readings, taken 2–3 times before and after different concentrations of salt, was calculated.

### 4.3. Hematocrit (HCT) Measurement

The submandibular vein was punctured using a 5.5 mm Goldenrod animal lancet (Medipoint Inc, Mineola, NY, USA). Whole blood samples were collected by using a Heparinized Microhematocrit Capillary Tube (Produce number: 22-362-566, Fisherbrand, Pittsburgh, PA, USA) and then centrifuged for 5 min in a Unico Micro-Hematocrit Centrifuge (Model: C-MH30, Dayton, NJ, USA). 

### 4.4. Renal Na^+^ Handling Studies

Na^+^ and endogenous Li^+^ concentrations in the urine, as well as Na^+^ concentrations in plasma specimens, were determined by flame photometry (contrAA 300, Analytik Jena, Germany). The levels of creatinine and albumin in plasma and urine were assessed using a commercial enzymatic assay kit (cat#, 80350, Crystal Chem, Elk Grove Village, IL, USA) and an ELISA kit (cat#, 80630, Elk Grove Village, IL, USA), respectively. Creatinine clearance (Ccr), total Na^+^ and Li^+^ (UNa^+^V and ULi^+^V) and fractional Na^+^ excretion rates (FENa^+^) were calculated from the following formula:

Ccr (mL/min) = (Ucr × 24-h urine volume)/(Pcr × 24 × 60)
UNa^+^V (mEq/day) = UNa^+^ × urine flow rate × 24 × 60
ULi^+^V (μEq/day) = ULi^+^ × urine flow rate × 24 × 60
FENa^+^ (%) = 100 × [(UNa^+^ × Pcr)/(PNa^+^ × Ucr)]
FEH_2_O (%) = 100 × [(24 h urine volume/24 × 60)/Ccr]

where Ucr and Pcr are the concentrations of creatinine in urine and plasma, respectively. UNa^+^ and PNa^+^ represent the concentrations of Na^+^ in urine and plasma respectively. ULi^+^ is the concentration of Li in urine. V is urine flow rate in volume per time.

### 4.5. Western Blotting

Western blotting was done as previously described [11,12,16]. Briefly, kidney cortex tissues were lysed in lysis buffer (Nonidet P-40 buffer containing 1% Nonidet P-40, 0.25% Na deoxycholate, 50 mM NaCl, 50 mM HEPES, 10% glycerol (pH 7.4), 1 mM Na vanadate, 0.5 mM Na fluoride, 1 mM phenylmethanesulfonyl fluoride, and protease inhibitor cocktail (Sigma-Aldrich, ST. LOUIS, MO USA). Proteins were separated by SDS-PAGE and transferred onto a PVDF membrane. After membranes were blocked with 5% milk for 60 min, they were probed with various primary antibodies overnight at 4 °C, followed by incubation with secondary antibodies (anti-Mouse-HRP or anti-Rabbit-HRP) in 5% milk TBST for 1 h at room temperature, and visualized with enhanced chemiluminescence reagent (PerkinElmer, Waltham, MA, USA). Antibodies for detection of p-ERK1/2 (Product number: #4370S), t-ERK1/2 (Product number: 9102) were obtained from Cell Signaling (Danvers, MA, USA_). Antibodies for detection of p-Src (Product number: 44660G), t-Src (Product number: sc-8056), HO-1 (Product number: ADI-SPA-895-F9121), and DNP (Product number: D9656) were obtained from Invitrogen (Camarillo, CA, USA), Santa Cruz (Santa Cruz, CA, USA), CA, Enzo (Farmingdale, NY, USA), and Sigma-Aldrich (ST. LOUIS, MO, USA), respectively. 

### 4.6. Histopathological Staining

Masson’s trichrome staining was performed on right kidney tissues by Wax-it Histology Services Inc. (Vancouver, BC V6T1Z3, CA). Computer-aided morphometry was used to quantify the percent area of fibrosis as previously reported [49,66]. 

### 4.7. Measurements of mRNA Expression (Quantitative Real-Time qRT-PCR)

For quantification of mRNA levels, the right kidney was used. Total RNA was purified from frozen kidney using Qiagen RNeasy Mini Kit as recommended by the manufacturer (Cat. 74104, Qiagen, Hilden, Germany). Isolated total RNA was used for qRT-PCR. The quantity and quality of the total RNA product were checked using the Nanodrop 2000/2000c spectrophotometer. One microgram RNA was used for cDNA synthesis in a reaction volume of 20 μL using the reverse transcription of RNA with the SuperScript III First-Strand Synthesis SuperMix for qRT-PCR (REF 11752-250, Invitrogen).

The primers used in this study were as follows: HO-1 forward, 5′-TGACACCTGAGGTCAAGCAC -3; reverse, 5′-GGCAGTATCTTGCACCAGGC-3; glutathione S-transferase M1 (GSTm1): forward, 5′- CTGACTTTGAGAAGCAGAAGCC -3′, reverse, 5′-TAGGTGTTGCGATGTAGCGG -3′; glutamate-cysteine ligases (GCLC) forward, 5′- CCCGGGCTGTCAACCG -3′, reverse, 5′-TACTCCACCTCGTCACCCC -3′; thioredoxin reductase 1 (TXNRD1) forward, 5′- AGCTGGTGGTTTCACCTTCC -3′, reverse, 5′-TTTTTGTTCGGCTTCAGGGC -3′; Actin forward, 5′- GGCTGTATTCCCCTCCATCG -3′, reverse, 5′-CCAGTTGGTAACAATGCCATGT -3′.

The LightCycler 480 PCR and detection system (Roche) was used for amplification and real-time quantification. The PCR reactions of each sample were performed in triplicate in a final volume of 20 μL in a 384 well plate. The PCR mixture contained 1 μL of 4X diluted cDNA template, 10 μL of SYBR Green Master Mix 2S and primers at a final concentration of 0.5 μM with the following conditions: initial denaturation at 95 °C for 5 min, followed by 45 amplification cycles at 95 °C for 15 s, and 60 °C for 30 s. After the amplification reaction melting curve analysis was performed, starting at 95 °C for 5 s, and 65 °C for 1 min, followed by cooling at 40 °C for 30 s. The comparative cycle threshold (Ct) method (also referred to as the DDCt Method) was used to analyze the data. Relative mRNA levels were calculated with the efficiency-corrected Ct method with β-actin as reference genes and mRNA levels for B6 NC as calibrator.

### 4.8. Statistics

Statistical data analysis was performed with GraphPad Prizm 7.01 (GraphPad Software, San Diego, CA, USA). We tested our data for normality using the Shapiro–Wilk test. Data that were normally distributed were compared using parametric methods. For data that failed the test for normality or data in which there were less than six observations, nonparametric methods were used for statistical comparisons. When parametric tests were used, we additionally checked for the robustness of our data by confirming the results with the nonparametric test. Data are represented as mean ±SEM, and error bars also indicate SEM *p*-values were calculated by (1) either unpaired two-tailed Students’ t-test or Mann–Whitney test for non-normal data or small sample size for directly comparing the means of two groups; (2) Two-way ANOVA followed by a Tukey’s multiple comparisons test for all the data sets analyzing the response of the two strains (B6 and TH) to two levels of salt intake (NC and HS). Throughout the study, a *p*-value of <0.05 using a two-tailed test was considered significant. 

## 5. Conclusions

What is new?

1. This study reports associations between renal Na/K-ATPase signaling-mediated Na^+^ excretion and blood pressure for the first time in polygenic obese TH mice.
2. Obese TH mice have normal proximal tubule Na^+^ handling that is likely driven by activated proximal Na/K-ATPase signaling-mediated natriuresis.
3. There is a relationship between dysfunctional Na/K-ATPase signaling and blunted pressure diuresis and natriuresis-induced hypertension in TH mice with a high salt diet.

What are the clinical implications?

1. TH mice appear to be a suitable mouse model of obesity and metabolic syndrome, offering insight into predisposing factors among individuals in susceptibility to renal injury due to obesity. For example, activated Na/K-ATPase signaling might be a potential predisposing factor.
2. When added to the evidence base from the obese TH mice, these results support the novel concept that Na/K-ATPase signaling-mediated Na^+^ handling is implicated in the pathophysiology of obesity and metabolic imbalance-associated hypertension.
3. This novel concept would promote the establishment of a new field of investigation for the etiology of hypertension, explicitly targeting the mechanisms of obesity-induced changes in ROS signaling and renal tubular cell transport.

## Figures and Tables

**Table 1:** Systolic blood pressure, total and fractional excretion of Na^+^ in C57BL/6J and TALLYHO/JngJ mice on normal chow.

	C57BL/6J (B6)	TALLYHO/JngJ (TH)
Systolic blood pressure, (mmHg, *n* = 8–10)	104.9 ± 1.6	110.5 ± 2.0
Total Na^+^ excretion rate UNa^+^V, mEq/day, *n* = 9–10)	0.619 ± 0.073	0.647 ± 0.065
Fractional Na^+^ excretion (FENa^+^, %, *n* = 9–10)	3.384 ± 0.324	3.875 ± 0.297
Value = Mean ± SEM		

**Table 2:** Food intake, Na^+^ intake, and water intake in C57BL/6J and TALLYHO/JngJ mice on normal and high salt diet.

Strain	Salt Diet	Group	Food Intake (g/mouse/day)	Na^+^ Intake (mEq/mouse/day)	Increases in 24 h Na^+^ Intake^Δ^	Water Intake (mL/mouse/day)
C57BL/6J (B6)	Normal chow (NC)	NC *n* = 9	6.644 ± 0.370	1.127 ± 0.063		8.428 ± 0.598
		HS *n* = 10	6.487 ± 0.431	1.100 ± 0.073		7.818 ± 0.388
	2% NaCl (7 days)	NC *n* = 9	6.210 ± 0.429	1.053 ± 0.073	−0.056 ± 0.175	8.357 ± 0.528
		HS *n* = 10	6.569 ± 0.350	2.285 ± 0.122 *	1.173 ± 0.627	8.424 ± 0.398
	4% NaCl (7 days)	NC *n* = 9	7.204 ± 0.383	1.222 ± 0.065	0.203 ± 0.306	8.356 ± 0.757
		HS *n* = 10	7.451 ± 0.555	5.183 ± 0.386 ***	1.311 ± 0.613	12.481 ± 0.531 ***
	8% NaCl (7 days)	NC *n* = 9	7.819 ± 0.567	1.326 ± 0.096	0.082 ± 0.147	10.427 ± 1.054
		HS *n* = 10	8.355 ± 0.596	11.624 ± 0.930 ***	1.316 ± 0.562	21.548 ± 1.563 ***
TALLYHO/JngJ (TH)	Normal chow (NC)	NC *n* = 8	6.105 ± 0.692	1.035 ± 0.117		6.178 ± 0.565
		HS *n* = 8	6.065 ± 0.431	1.028 ± 0.073		6.540 ± 0.318
	2% NaCl (7 days)	NC *n* = 8	5.748 ± 0.263	0.975 ± 0.045	0.004 ± 0.250	7.218 ± 0.287
		HS *n* = 8	4.588 ± 0.168	1.596 ± 0.058	0.607 ± 0.366 ^#^	5.553 ± 0.443
	4% NaCl (7 days)	NC *n* = 8	5.816 ± 0.295	0.986 ± 0.050	0.203 ± 0.306	7.509 ± 0.279
		HS *n* = 8	5.041 ± 0.113	3.507 ± 0.079 ***	1.218 ± 0.260	7.826 ± 0.321
	8% NaCl (7 days)	NC *n* = 8	7.265 ± 0.481	1.232 ± 0.082	0.262 ± 0.227	7.509 ± 0.279
		HS *n* = 8	6.350 ± 0.620	8.835 ± 0.863 ***	1.541 ± 0.759	16.474 ± 1.127 ***

Normal chow (0.39% Sodium): HS, high salt. Value = Mean ± SEM; *** *p* < 0.001, * *p* < 0.05 vs. B6 NC or TH NC respectively; ^#^ *p* < 0.05 vs. B6 HS. Δ, self-comparison, Na^+^ intake value at 2%, 4%, and 8% NaCl is subtracted by the previous value at NC, 2%, and 4% NaCl, respectively, which is then divided by the previous Na^+^ intake value.

## Supplementary Materials

Supplementary materials can be found at https://www.mdpi.com/1422-0067/20/14/3495/s1.

## Abbreviations

ROS	Reactive oxygen species
TH	TALLYHO/JngJ mouse
B6	C57BL/6J mouse
HS	High salt diet
NC	Normal chow
BP	Blood pressure
DR	Dahl salt-resistant rat
DS	Dahl salt-sensitive rat
WT	Wild-type
ULi+V	Urinary lithium excretion rate
UNa+V & PNa+	Urinary Na+ excretion rate and plasma Na+ concentration
FENa+	Fractional Na+ excretion
Ccr	Creatinine clearance
HCT	Hematocrit
CKD	Chronic kidney disease
CTS	Cardiotonic steroids

## References

[1] Ng M., Fleming T., Robinson M., Thomson B., Graetz N., Margono C., Mullany E.C., Biryukov S., Abbafati C., Abera S.F. (2014). Global, regional, and national prevalence of overweight and obesity in children and adults during 1980–2013: A systematic analysis for the global burden of disease study 2013. Lancet.

[2] Chen J., Gu D., Huang J., Rao D.C., Jaquish C.E., Hixson J.E., Chen C.S., Chen J., Lu F., Hu D. (2009). Metabolic syndrome and salt sensitivity of blood pressure in non-diabetic people in china: A dietary intervention study. Lancet.

[3] Hall M.E., do Carmo J.M., da Silva A.A., Juncos L.A., Wang Z., Hall J.E. (2014). Obesity, hypertension, and chronic kidney disease. Int. J. Nephrol. Renov. Dis..

[4] Nizar J.M., Bhalla V. (2017). Molecular mechanisms of sodium-sensitive hypertension in the metabolic syndrome. Curr. Hypertens. Rep..

[5] Guyton A.C. (1961). Physiologic regulation of arterial pressure. Am. J. Cardiol..

[6] Guyton A.C., Coleman T.G. (1999). Quantitative analysis of the pathophysiology of hypertension. 1969. J. Am. Soc. Nephrol..

[7] Guyton A.C. (1992). Kidneys and fluids in pressure regulation. Small volume but large pressure changes. Hypertension.

[8] Hall J.E. (2016). Renal dysfunction, rather than nonrenal vascular dysfunction, mediates salt-induced hypertension. Circulation.

[9] Morris R.C., Schmidlin O., Sebastian A., Tanaka M., Kurtz T.W. (2016). Vasodysfunction that involves renal vasodysfunction, not abnormally increased renal retention of sodium, accounts for the initiation of salt-induced hypertension. Circulation.

[10] Skou J.C. (1957). The influence of some cations on an adenosine triphosphatase from peripheral nerves. Biochim. Biophys. Acta.

[11] Yan Y., Shapiro A.P., Haller S., Katragadda V., Liu L., Tian J., Basrur V., Malhotra D., Xie Z.J., Abraham N.G. (2013). Involvement of reactive oxygen species in a feed-forward mechanism of na/k-atpase-mediated signaling transduction. J. Biol. Chem..

[12] Liu J., Yan Y., Liu L., Xie Z., Malhotra D., Joe B., Shapiro J.I. (2011). Impairment of na/k-atpase signaling in renal proximal tubule contributes to dahl salt-sensitive hypertension. J. Biol. Chem..

[13] Yan Y., Haller S., Shapiro A., Malhotra N., Tian J., Xie Z., Malhotra D., Shapiro J.I., Liu J. (2012). Ouabain-stimulated trafficking regulation of the na/k-atpase and nhe3 in renal proximal tubule cells. Mol. Cell. Biochem..

[14] Kim J.H., Saxton A.M. (2012). The tallyho mouse as a model of human type 2 diabetes. Methods Mol. Biol..

[15] Periyasamy S.M., Liu J., Tanta F., Kabak B., Wakefield B., Malhotra D., Kennedy D.J., Nadoor A., Fedorova O.V., Gunning W. (2005). Salt loading induces redistribution of the plasmalemmal na/k-atpase in proximal tubule cells. Kidney Int..

[16] Yan Y., Shapiro A.P., Mopidevi B.R., Chaudhry M.A., Maxwell K., Haller S.T., Drummond C.A., Kennedy D.J., Tian J., Malhotra D. (2016). Protein carbonylation of an amino acid residue of the na/k-atpase alpha1 subunit determines na/k-atpase signaling and sodium transport in renal proximal tubular cells. J Am. Heart. Assoc..

[17] Gupta S., Yan Y., Malhotra D., Liu J., Xie Z., Najjar S.M., Shapiro J.I. (2012). Ouabain and insulin induce sodium pump endocytosis in renal epithelium. Hypertension.

[18] Didion S.P., Lynch C.M., Faraci F.M. (2007). Cerebral vascular dysfunction in tallyho mice: A new model of type ii diabetes. Am. J. Physiol. Heart Circ. Physiol..

[19] O’Connor W.J. (1977). Normal sodium balance in dogs and in man. Cardiovasc. Res..

[20] Gupta B.N., Linden R.J., Mary D.A., Weatherill D. (1981). The influence of high and low sodium intake on blood volume in the dog. Q. J. Exp. Physiol..

[21] Grant H., Reischsman F. (1946). The effects of the ingestion of large amounts of sodium chloride on the arterial and venous pressures of normal subjects. Am. Heart J..

[22] Eckel R.H., Grundy S.M., Zimmet P.Z. (2005). The metabolic syndrome. Lancet.

[23] Mandal R., Loeffler A.G., Salamat S., Fritsch M.K. (2012). Organ weight changes associated with body mass index determined from a medical autopsy population. Am. J. Forensic Med. Pathol..

[24] Kasiske B.L., Napier J. (1985). Glomerular sclerosis in patients with massive obesity. Am. J. Nephrol..

[25] Tsuboi N., Okabayashi Y., Shimizu A., Yokoo T. (2017). The renal pathology of obesity. Kidney Int. Rep..

[26] (2017). National Chronic Kidney Disease Fact Sheet, 2017.

[27] Expert Panel on Detection, Evaluation, and Treatment of High Blood Cholesterol in Adults (2001). Executive summary of the third report of the national cholesterol education program (ncep) expert panel on detection, evaluation, and treatment of high blood cholesterol in adults (adult treatment panel iii). JAMA.

[28] Alberti K.G., Zimmet P., Shaw J., IDF Epidemiology Task Force Consensus Group (2005). The metabolic syndrome—A new worldwide definition. Lancet.

[29] Foster M.C., Hwang S.J., Porter S.A., Massaro J.M., Hoffmann U., Fox C.S. (2011). Fatty kidney, hypertension, and chronic kidney disease: The framingham heart study. Hypertension.

[30] Denvir J., Boskovic G., Fan J., Primerano D.A., Parkman J.K., Kim J.H. (2016). Whole genome sequence analysis of the tallyho/jng mouse. BMC Genom..

[31] Parkman J.K., Denvir J., Mao X., Dillon K.D., Romero S., Saxton A.M., Kim J.H. (2017). Congenic mice demonstrate the presence of qtls conferring obesity and hypercholesterolemia on chromosome 1 in the tallyho mouse. Mamm. Genome.

[32] Creecy A., Uppuganti S., Unal M., Clay Bunn R., Voziyan P., Nyman J.S. (2018). Low bone toughness in the tallyho model of juvenile type 2 diabetes does not worsen with age. Bone.

[33] Trevisan R., Bruttomesso D., Vedovato M., Brocco S., Pianta A., Mazzon C., Girardi C., Jori E., Semplicini A., Tiengo A. (1998). Enhanced responsiveness of blood pressure to sodium intake and to angiotensin ii is associated with insulin resistance in iddm patients with microalbuminuria. Diabetes.

[34] Iuchi H., Sakamoto M., Suzuki H., Kayama Y., Ohashi K., Hayashi T., Ishizawa S., Yokota T., Tojo K., Yoshimura M. (2016). Effect of one-week salt restriction on blood pressure variability in hypertensive patients with type 2 diabetes. PLoS ONE.

[35] Hall J.E. (2003). The kidney, hypertension, and obesity. Hypertension.

[36] Hall J.E. (1997). Mechanisms of abnormal renal sodium handling in obesity hypertension. Am. J. Hypertens..

[37] Kassab S., Kato T., Wilkins F.C., Chen R., Hall J.E., Granger J.P. (1995). Renal denervation attenuates the sodium retention and hypertension associated with obesity. Hypertension.

[38] Wehner P., Shapiro J.I. (2014). Renal denervation to treat cardiac fibrosis?. J. Am. Heart. Assoc..

[39] Bhatt D.L., Kandzari D.E., O’Neill W.W., D’Agostino R., Flack J.M., Katzen B.T., Leon M.B., Liu M., Mauri L., Negoita M. (2014). A controlled trial of renal denervation for resistant hypertension. N. Engl. J. Med..

[40] Engholm M., Bertelsen J.B., Mathiassen O.N., Botker H.E., Vase H., Peters C.D., Bech J.N., Buus N.H., Schroeder A.P., Rickers H. (2018). Effects of renal denervation on coronary flow reserve and forearm dilation capacity in patients with treatment-resistant hypertension. A randomized, double-blinded, sham-controlled clinical trial. Int. J. Cardiol..

[41] Bhat A., Kuang Y.M., Gan G.C., Burgess D., Denniss A.R. (2015). An update on renal artery denervation and its clinical impact on hypertensive disease. BioMed Res. Int..

[42] Wilcox C.S. (2002). Reactive oxygen species: Roles in blood pressure and kidney function. Curr. Hypertens. Rep..

[43] Ando K. (2013). Increased salt sensitivity in obese hypertension: Role of the sympathetic nervous system. Curr. Hypertens. Rev..

[44] Hubens L.E., Verloop W.L., Joles J.A., Blankestijn P.J., Voskuil M. (2013). Ischemia and reactive oxygen species in sympathetic hyperactivity states: A vicious cycle that can be interrupted by renal denervation?. Curr. Hypertens. Rep..

[45] Liu J., Tian J., Haas M., Shapiro J.I., Askari A., Xie Z. (2000). Ouabain interaction with cardiac Na+/K+-ATPase initiates signal cascades independent of changes in intracellular na+ and ca2+ concentrations. J. Biol. Chem..

[46] Liu L., Li J., Liu J., Yuan Z., Pierre S.V., Qu W., Zhao X., Xie Z. (2006). Involvement of Na+/K+-ATPase in hydrogen peroxide-induced hypertrophy in cardiac myocytes. Free Radic. Biol. Med..

[47] Xie Z., Kometiani P., Liu J., Li J., Shapiro J.I., Askari A. (1999). Intracellular reactive oxygen species mediate the linkage of Na+/K+-ATPase to hypertrophy and its marker genes in cardiac myocytes. J. Biol. Chem..

[48] Tian J., Liu J., Garlid K.D., Shapiro J.I., Xie Z. (2003). Involvement of mitogen-activated protein kinases and reactive oxygen species in the inotropic action of ouabain on cardiac myocytes. A potential role for mitochondrial k(atp) channels. Mol. Cell. Biochem..

[49] Kennedy D.J., Vetteth S., Periyasamy S.M., Kanj M., Fedorova L., Khouri S., Kahaleh M.B., Xie Z., Malhotra D., Kolodkin N.I. (2006). Central role for the cardiotonic steroid marinobufagenin in the pathogenesis of experimental uremic cardiomyopathy. Hypertension.

[50] Elkareh J., Kennedy D.J., Yashaswi B., Vetteth S., Shidyak A., Kim E.G., Smaili S., Periyasamy S.M., Hariri I.M., Fedorova L. (2007). Marinobufagenin stimulates fibroblast collagen production and causes fibrosis in experimental uremic cardiomyopathy. Hypertension.

[51] Yan Y., Shapiro J.I. (2016). The physiological and clinical importance of sodium potassium atpase in cardiovascular diseases. Curr. Opin. Pharmacol..

[52] Sodhi K., Maxwell K., Yan Y., Liu J., Chaudhry M.A., Getty M., Xie Z., Abraham N.G., Shapiro J.I. (2015). Pnaktide inhibits na/k-atpase reactive oxygen species amplification and attenuates adipogenesis. Sci. Adv..

[53] Sodhi K., Srikanthan K., Goguet-Rubio P., Nichols A., Mallick A., Nawab A., Martin R., Shah P.T., Chaudhry M., Sigdel S. (2017). pNaKtide attenuates steatohepatitis and atherosclerosis by blocking Na/K-ATPase/ROS amplification in c57bl6 and apoe knockout mice fed a western diet. Sci. Rep..

[54] Liu J., Tian J., Chaudhry M., Maxwell K., Yan Y., Wang X., Shah P.T., Khawaja A.A., Martin R., Robinette T.J. (2016). Attenuation of Na/K-ATPase mediated oxidant amplification with pnaktide ameliorates experimental uremic cardiomyopathy. Sci. Rep..

[55] Petrushanko I.Y., Mitkevich V.A., Lakunina V.A., Anashkina A.A., Spirin P.V., Rubtsov P.M., Prassolov V.S., Bogdanov N.B., Hanggi P., Fuller W. (2017). Cysteine residues 244 and 458-459 within the catalytic subunit of na,k-atpase control the enzyme’s hydrolytic and signaling function under hypoxic conditions. Redox Biol..

[56] Petrushanko I., Bogdanov N., Bulygina E., Grenacher B., Leinsoo T., Boldyrev A., Gassmann M., Bogdanova A. (2006). Na-K-ATPase in rat cerebellar granule cells is redox sensitive. Am. J. Physiol. Regul. Integr. Comp. Physiol..

[57] Liu J., Yan Y., Nie Y., Shapiro J.I. (2017). Na/K-ATPase signaling and salt sensitivity: The role of oxidative stress. Antioxidants.

[58] Lakunina V.A., Burnysheva K.M., Mitkevich V.A., Makarov A.A., Petrushanko I.Y. (2017). changes in the receptor function of na,k-atpase during hypoxia and ischemia. Mol. Biol..

[59] Cwynar M., Stompor T., Barton H., Grodzicki T. (2014). Endogenous lithium clearance: A diagnostic method of assessing sodium sensitivity in hypertension. Methodological and clinical implications. Kardiol. Pol..

[60] Venezia A., Barba G., Russo O., Capasso C., De Luca V., Farinaro E., Cappuccio F.P., Galletti F., Rossi G., Strazzullo P. (2010). Dietary sodium intake in a sample of adult male population in southern italy: Results of the olivetti heart study. Eur. J. Clin. Nutr..

[61] Strazzullo P., Barba G., Cappuccio F.P., Siani A., Trevisan M., Farinaro E., Pagano E., Barbato A., Iacone R., Galletti F. (2001). Altered renal sodium handling in men with abdominal adiposity: A link to hypertension. J. Hypertens..

[62] Bagrov A.Y., Shapiro J.I., Fedorova O.V. (2009). Endogenous cardiotonic steroids: Physiology, pharmacology, and novel therapeutic targets. Pharm. Rev..

[63] Koomans H.A., Boer W.H., Dorhout Mees E.J. (1989). Evaluation of lithium clearance as a marker of proximal tubule sodium handling. Kidney Int..

[64] Liang M., Cai T., Tian J., Qu W., Xie Z.J. (2006). Functional characterization of src-interacting Na/K-ATPase using RNA interference assay. J. Biol. Chem..

[65] Heppner D.E., Dustin C.M., Liao C., Hristova M., Veith C., Little A.C., Ahlers B.A., White S.L., Deng B., Lam Y.W. (2018). Direct cysteine sulfenylation drives activation of the src kinase. Nat. Commun..

[66] Haller S.T., Yan Y., Drummond C.A., Xie J., Tian J., Kennedy D.J., Shilova V.Y., Xie Z., Liu J., Cooper C.J. (2016). Rapamycin attenuates cardiac fibrosis in experimental uremic cardiomyopathy by reducing marinobufagenin levels and inhibiting downstream pro-fibrotic signaling. J. Am. Heart. Assoc..

