# Supplementary material for: Metabolic Syndrome and Salt-Sensitive Hypertension in Polygenic Obese TALLYHO/JngJ Mice: Role of Na/K-ATPase Signaling
Source: Int J Mol Sci. 2019 Jul 16;20(14):3495. doi: 10.3390/ijms20143495 (PMC6678942; doi:10.3390/ijms20143495)
Supplement: Supplementary file 1 [file ijms-20-03495-s001.pdf]

Supplementary Materials

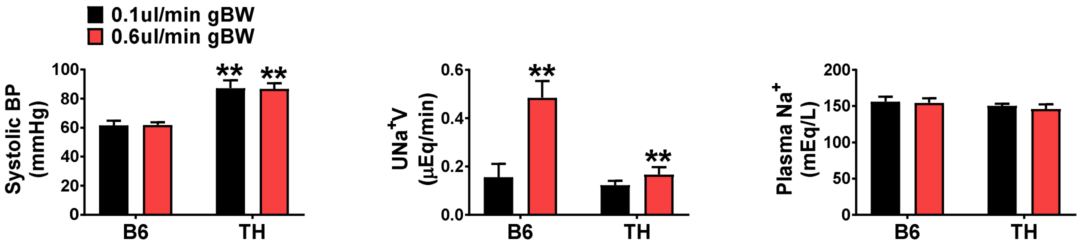

Figure S1. Effect of a Na^+ bolus and volume expansion on systolic blood pressure (SBP, left), absolute Na^+ excretion rate (UNa^+V, middle), and Plasma Na^+ (right). Isotonic saline was infused at the rate of 0.1 µL/min g body weight (BW). After a 60 min equilibration period, a 60 min urine collection was obtained. A separate group of mice was prepared as described above, but the intravenous infusion rate was increased to 0.6 µL/min g BW after the equilibration period. The TH mice exhibited significantly higher SBP, but reduced total sodium excretion compared with the B6 mice. No significant change in plasma Na^+ was observed in B6 and TH mice during the experimental periods. n = 4–7. ** $p < 0.001$ versus B6 0.1 µL/min g BW. # versus TH 0.1 µL/min g BW.

Supplemental Experimental Procedures

Adult male mice were used throughout and were obtained from the Jackson Laboratory (Bar Harbor, Maine). On the day of the experiment, B6 and TH mice weighing 31–36 g and 40–46 g were anesthetized with ketamine (50 µg/g body weight, intraperitoneally) and thiobutabarbital (Inactin, 100 µg/g body weight, intraperitoneally, Sigma-Aldrich, ST. LOUIS, MO) [1]. Animals were placed on a thermostatically controlled pad set to maintain body temperature at 37 °C (FHC, Bowdoinham, ME).

The right jugular vein was catheterized with PE-10 tubing for the administration of isotonic saline. The bladder was catheterized with PE-50 tubing via a suprapubic incision for the collection of urines [1–3]. Systolic blood pressure was monitored by a tail-cuff method using CODA 8-Channel High Throughout Non-Invasive Blood Pressure System (Kent Scientific, Boston, MA). Na^+ concentration was determined by a flame photometer (contrAA 300, Analytik Jena, Germany).

References

1. Loreaux, E.L.; Kaul, B.; Lorenz, J.N.; Lingrel, J.B. Ouabain-sensitive alpha1 na,k-atpase enhances natriuretic response to saline load. *J. Am. Soc. Nephrol.* **2008**, *19*, 1947–1954.
2. Cervenka, L.; Mitchell, K.D.; Navar, L.G. Renal function in mice: Effects of volume expansion and angiotensin ii. *J. Am. Soc. Nephrol.* **1999**, *10*, 2631–2636.
3. Kibble, J.D.; Balloch, K.J.; Neal, A.M.; Hill, C.; White, S.; Robson, L.; Green, R.; Taylor, C.J. Renal proximal tubule function is preserved in cftr(tm2cam) deltaf508 cystic fibrosis mice. *J. Physiol.* **2001**, *532*, 449–457.

© 2019 by the authors. Submitted for possible open access publication under the terms and conditions of the Creative Commons Attribution (CC BY) license (http://creativecommons.org/licenses/by/4.0/).
